# Interdisciplinary CBT treatment for patients with odontophobia and dental anxiety related to psychological trauma experiences: a case series

**DOI:** 10.1186/s12888-024-06055-w

**Published:** 2024-09-10

**Authors:** Yngvill Ane Stokke Westad, Gina Løge Flemmen, Stian Solem, Trine Monsen, Henriette Hollingen, Astrid Feuerherm, Audun Havnen, Kristen Hagen

**Affiliations:** 1 Molde Competence Clinic for Public Dental Health Service, Møre and Romsdal County Authority, Molde, Norway; 2 Department of Psychology, Norwegian University of Science and Technology, Trondheim, Norway; 3 Center for Oral Health Services and Research, Mid-Norway (TkMidt), Trondheim, Norway; 4 Nidaros Division of Psychiatry, Community Mental Health Centre, St. Olav’s University Hospital, Trondheim, Norway; 5 Molde Hospital, Møre og Romsdal Hospital Trust, Molde, Norway; 6 Bergen Center for Brain Plasticity, Haukeland University Hospital, Bergen, Norway; 7 Department of Mental Health, Norwegian University of Science and Technology, Trondheim, Norway

**Keywords:** Odontophobia, Dental anxiety, Psychological trauma, PTSD, Oral health, Oral health, CBT, Interdisciplinary treatment

## Abstract

**Background:**

While cognitive-behavioural therapy (CBT) is a well-established treatment for odontophobia, research is sparse regarding its effect on patients with dental anxiety related to psychological trauma experiences. This study aimed to evaluate changes in symptoms and acceptability of interdisciplinary Torture, Abuse, and Dental Anxiety (TADA) team treatment for patients with odontophobia or dental anxiety. We also wanted to describe the sample’s oral health status. The TADA teams offer targeted anxiety treatment and adapted dental treatment using a CBT approach.

**Methods:**

The study used a naturalistic, case series design and included 20 consecutively referred outpatients at a public TADA dental clinic. Pre- and post-treatment assessments included questionnaires related to the degree of dental anxiety, post-traumatic stress, generalized anxiety, and depression. Patients underwent a panoramic X-ray before treatment. Before dental restoration, patients underwent an oral health examination to determine the mucosal and plaque score (MPS) and the total number of decayed, missing, and filled teeth (DMFT). Patients were referred to dentist teams for further dental treatment and rehabilitation (phase 2) after completing CBT in the TADA team (Phase 1). Results from the dental treatment in phase 2 is not included in this study.

**Results:**

All patients completed the CBT treatment. There were significant improvements in symptoms of dental anxiety, post-traumatic stress, and depression and moderate changes in symptoms of generalized anxiety. Dental statuses were heterogeneous in terms of the severity and accumulated dental treatment needs. The TADA population represented the lower socioeconomic range; 15% of patients had higher education levels, and half received social security benefits. All patients were referred to and started adapted dental treatment (phase 2).

**Conclusions:**

TADA treatment approach appears acceptable and potentially beneficial for patients with odontophobia and dental anxiety related to psychological trauma experiences. The findings suggest that further research, including larger controlled studies, is warranted to validate these preliminary outcomes.

**Trial registration:**

The study was approved by the regional ethical committee in Norway (REK-Midt: 488462) and by the Data Protection Board at Møre and Romsdal County Authority.

**Supplementary Information:**

The online version contains supplementary material available at 10.1186/s12888-024-06055-w.

## Background

Patients with mental disorders have a greater risk of oral and dental diseases than the general population. Psychiatric diagnoses are associated with poor dental status, such as carious, missing or filled teeth or surfaces [1], and patients with severe mental illness are almost three times more likely to lose all of their teeth compared to the general population [2]. This may be caused by several individual or cumulative factors, such as the inability to perform self-care, diet and lifestyle factors, difficulties in accessing health care services, poor economic status, a negative attitude towards health care providers, shame and anxiety, difficulties cooperating with treatment, and drug use and drug treatment side effects [1, 3–6].

Patients referred for dental anxiety treatment have moderately high levels of comorbid psychological conditions [7], and this patient group differs with respect to the age of onset, origins, and manifestations [8]. Individuals with high dental anxiety report more mental health symptoms, poorer oral health, more avoidance behaviour, and more irregular dental visits than those with no or low anxiety [9–12]. Furthermore, large variations in oral health and dental treatment needs have been found in patients with dental anxiety and phobia [13, 14].

Patients with anxiety disorders, especially post-traumatic stress disorder (PTSD), could be especially prone to developing fears of dental treatment [15]. The study found that 42.0% of patients with PTSD reported high dental anxiety, compared to 17.6–31.3% in other psychiatric groups, and 4.2% in healthy controls [15]. Approximately 20% of female patients seeking dental care may have encountered childhood sexual abuse [16]. Patients who have experienced traumatic events may exhibit distinct psychological and emotional responses that can complicate the treatment process [16–18]. Furthermore, elements of abuse can resemble the dental treatment environment and make it difficult to tolerate dental treatment [17, 19, 20]. This suggests that it is important for treatment and professionals to be considerate of the patient’s trauma history [21, 22].

In 2010, the Norwegian Department of Health concluded that patients who were exposed to torture, sexual abuse, and/or violence in close relationships and/or had odontophobia had inadequate treatment options in the Norwegian public oral health care service [23]. Based on an overriding goal of ensuring equal access to oral health services regardless of ethnic background, sex, personal finances, and life situations, it was decided to establish interdisciplinary “Torture, Abuse and Dental Anxiety (TADA) teams” nationally. These teams consist of both clinical psychologists and oral health professionals. TADA teams offer anxiety treatment and/or adapted dental treatment based on cognitive-behavioural therapy (CBT) principles.

Previous studies have showed promising results regarding the effectiveness of CBT for odontophobia [24–26]. However, there is a lack of studies specifically evaluating CBT for patients with odontophobia and dental anxiety who have been exposed to sexual abuse, violence in close relationships, or torture. To our knowledge, there is not any published studies on the effect of dental anxiety treatment in patients with post-traumatic stress symptoms related to abuse or torture in their literature review. However, we found one study that reported an effect of CBT treatment on dental anxiety in patients with post-traumatic stress symptoms triggered by previous dental treatment [27]. It is uncertain whether findings from that study could be generalized to patients with more extensive and severe trauma experiences originating from torture, abuse, or violence in close relationships.

The aim of this study was therefore to evaluate the change in symptoms from pre-treatment to post-treatment after integrated psychological and dental treatment for a vulnerable patient group who have been exposed to torture, sexual abuse, and/or violence in close relationships and/or who have odontophobia, in a naturalistic case series design, This is important given that the implementation of TADA teams is unique, and the service has not been evaluated [28].

## Methods

### Participants and procedure

A naturalistic case series design was used. The inclusion criteria for the TADA treatment were: (a) confirming a history of being subjected to torture, abuse, and/or violence in close relationships and/or confirming clinical symptoms of odontophobia (including blood/injection/injury- phobias), (b) being aged 21 years or more at the point of orientation, (c) being willing and having the ability to commit to a treatment plan prepared in collaboration with an interdisciplinary treatment team, and (d) understanding the rationale and treatment principles for the relevant course of treatment. The exclusion criteria were patients who: (a) had an organic disorder such as dementia, delirium, or severe memory problems, or suffered from a severe depressive disorder, mania, or ongoing psychosis at the time of evaluation, and (b) had known cognitive/language delays corresponding to an intellectual disability and were not considered to be able to benefit from the treatment approach because of this.

Patients were invited to the TADA clinic for an orientation with a clinical psychologist (1–2 appointments) after referral. During the orientation, the motivation to commit to therapy was addressed (e.g., willingness to meet at regular intervals for CBT treatment appointments and to gradually expose themselves to feared events). At the time of orientation, patients who confirmed having dental treatment difficulties (e.g., did not seek dental treatment, failed to carry out dental treatment, and/or endured dental treatment with great difficulty), and/or being exposed to sexual abuse/violence/torture, and were willing to commit to CBT treatment, underwent a diagnostic evaluation and were accepted into the TADA treatment program.

After interdisciplinary CBT treatment (phase 1), patients were referred by the first TADA team to the second TADA team (phase 2). Patients referred to the second TADA team were required to attend their first appointment unaccompanied. The first meeting involved reviewing discharge summaries from the first TADA team and developing a treatment plan for dental restoration. The second TADA dentist team (Phase 2) did not function as CBT therapists in this study. If patients did not need full-scale interdisciplinary CBT treatment at the point of orientation, they were referred directly to a TADA dentist team for adapted dental treatment. If needed, the TADA team referred patients to emergency dental treatment before or after the CBT intervention. Both interdisciplinary CBT treatment and dental treatment were delivered free of charge. The TADA dentist and dental nurse involved in phase 1 have their CBT training from continuous guidance and working in collaboration with the CBT trained psychologist. The TADA team involved in phase 2 consist of another dentist and dental nurse with basic training in CBT provided by the TADA psychologist. Both TADA teams participate in annual courses to maintain basic skills in CBT.

Prior to treatment initiation, dental anxiety was assessed with the specific phobia disorder module of the Mini International Neuropsychiatric Interview (MINI) version 7.0.2. [29] and dental fear and anxiety symptom questionnaires. Patients exposed to torture, sexual abuse, or violence in close relationships were included in the study regardless of whether the diagnostic criteria for odontophobia were met. These patients were further assessed with questionnaires assessing exposure to potentially stressful life events [30] and related posttraumatic stress symptom severity [31]. The patients answered their highest level of education completed (primary school, upper secondary school, college/university up to 5 years, or college/university over 5 years). Patients with college/university experience were defined as “higher education”. Furthermore, patients answered their current marital status (single, cohabiting/married, or in a relationship, but not cohabiting). The degree to which personal economy status had affected dental treatment execution was answered as either “not at all”, “to some extent” or “to a large extent”.

### Measures

The Modified Dental Anxiety Scale (MDAS) [32] is a brief, self-administered questionnaire consisting of five questions regarding different dentist treatment situations. Each item is scored on a Likert scale ranging from “1” (not anxious) to “5” (extremely anxious). The item scores are summed to produce a total score ranging from 5 to 25. A cut-off score of 19 indicates high dental anxiety [33, 34].

The Dental Fear Survey (DFS) [35, 36] is a brief measure of dental anxiety and fear that consists of 20 items. Each item is scored on a Likert scale from “1” (never or not at all) to “5” (always or very much). Total DFS scores range from 20 to 100, with increasing scores indicating higher levels of fear. A total score of 20 indicates “no fear,” a score of 21–40 indicates low fear, a score of 41–79 indicates moderate fear, and a score of 80–100 indicates high fear [35, 36].

The Stressful Life Events Screening Questionnaire (SLESQ) [30, 37] is a 13-item questionnaire assessing lifetime exposure to various traumatic experiences. Each item represents different traumatic experiences and is scored as either “yes” or “no” depending on whether the individual has been exposed to the incident. This questionnaire was used exclusively at pretreatment to screen for exposure to potential traumatic experiences.

The PTSD Checklist for the DSM-5 (PCL-5) [31] is a 20-item questionnaire assessing 20 PTSD criteria outlined in the Diagnostic and Statistical Manual of Mental Disorders-Fifth Edition (DSM-5). Each item is scored on a Likert scale ranging from “0” (not at all) to “4” (extremely) based on the occurrence of symptoms during the last month. A total cut-off score of 33 has been found to efficiently detect PTSD [38]. Only patients who were confirmed to have been exposed to potentially traumatic life events completed the PCL-5.

The Patient Health Questionnaire-9 (PHQ-9) [39] consists of nine items measuring depressive symptoms. Each of the nine DSM-IV criteria is scored on a Likert scale ranging from “0” (not at all) to “3” (nearly every day) with total scores ranging from 0 to 27, with higher scores reflecting greater depression severity. PHQ-9 scores of 5, 10, 15, and 20 represent mild, moderate, moderately severe, and severe depression, respectively.

The Generalized Anxiety Disorder-7 (GAD-7) [40] is a brief measure for assessing symptoms of generalized anxiety disorder. The measure consists of seven items measuring worry and anxiety symptoms. Each item is scored on a Likert scale, ranging from “0” (not at all) to “3” (nearly every day). A total score above 10 is considered to be within the clinical range. The GAD-7 is also a measure of anxiety symptoms in general [41].

The mucosal plaque score (MPS) [42] is designed to evaluate oral health and oral hygiene. The index consists of two measures: a four-point mucosal score (MS) and a four-point plaque score (PS). The scores are combined, and the total score ranges from 2 to 8, with higher scores indicating poorer oral health and oral hygiene.

The decayed, missing, and filled teeth index (DMFT) quantifies a person’s total number of untreated decayed, missing, and filled teeth and is commonly used in oral epidemiology to quantify the extent of caries [43]. “Decayed” corresponds to primary or secondary caries in dentin, while “Missing” and “Filled” correspond to missing teeth due to caries, root residues/carious teeth beyond repair and filled/restored teeth with no sign of caries in dentin, respectively. 3rd molars were excluded from the DMFT evaluation, except in situations where these functioned as second molars. The index is frequently used to evaluate and monitor oral health and in oral health interventions [44, 45].

#### Oral health and dental status examinations

Before interdisciplinary CBT treatment, the TADA patients underwent a panoramic X-ray (orthopantomography; OPG). OPG provides a panoramic single radiograph image of the teeth, maxilla, mandible, and adjacent tissue. OPG is a frequently employed radiological examination [46]. The TADA dentist conducted a dental status evaluation when the patients could tolerate the procedure. To evaluate a patient’s oral health status and dental treatment needs, the dentist determined their mucosal and plaque score (MPS) and the total number of decayed, missing, and filled teeth (DMFT).

#### CBT intervention (phase 1)

The TADA treatment consisted of two phases. In the first phase, patients were offered interdisciplinary CBT treatment before being referred to an other TADA dentist team for further dental treatment and rehabilitation (second phase).

The interdisciplinary CBT treatment team consisted of a dentist, a dental nurse, and a clinical psychologist delivering CBT together. During orientation to TADA treatment, a psychologist prepared the patient for CBT treatment by providing psychoeducation and rationale for exposure therapy, mapped catastrophic thoughts and safety and avoidance behaviours, examined the patient’s motivation for treatment, and clarified the treatment framework (e.g., treatment duration and structure, dental treatment clarification). The CBT treatment team then offered cognitive-behavioural treatment to challenge patients’ catastrophic thoughts and beliefs about dental treatment and find ways to adapt dental treatment to make it feasible. Patients with odontophobia or dental anxiety related to exposure to torture, sexual abuse, or violence in close relationships also received trauma-relevant psychoeducation and were taught skills on how to cope with trauma symptoms to facilitate new remedial learning experiences. The CBT intervention did not include trauma therapy directly focusing on the primary traumatic event. In addition to cognitive restructuring, in-vivo exposure therapies were conducted, tailored to maximize the disconfirmation of each patient’s unique catastrophic beliefs. While these exposure therapies varied somewhat among patients, the majority of CBT sessions included exposure to activities such as using dental mirrors, probes, polishing, administering anaesthesia, tartar cleaning, drilling, filling procedures, and, when necessary, the process of obtaining impressions and extracting root residues or teeth. Throughout the CBT phase, both dental healthcare professionals and a psychologist were typically present.

Anxiolytic drugs were not offered as part of the treatment intervention. The standard CBT treatment consisted of weekly therapy sessions (1–1.5 h) for up to 12 sessions. All exposure sessions were carried out in vivo in the dental office. The extent of the psychologist’s involvement during exposure sessions was evaluated on an individual basis. Additional sessions could be granted if the TADA team expected the patient to benefit from further follow-up.

#### Dental treatment intervention (phase 2)

Only limited dental treatment was carried out in the interdisciplinary CBT phase of the TADA treatment. In this phase, dental treatment was carried out only for the purpose of exposure and for facilitating new learning experiences. In case of acute infections and an immediate need for dental treatment before or during CBT treatment, patients were referred for dental treatment under general anaesthesia before further CBT treatment was provided. Two patients (10%) in this sample underwent dental treatment under general anaesthesia during the CBT intervention phase.

After interdisciplinary CBT treatment, patients were referred to a different TADA dentist team consisting of a dentist and a dental nurse for dental treatment and rehabilitation. This second phase of the treatment was not time limited. These TADA dentist teams were trained in CBT interventions but did not work collaboratively with a psychologist.

### Statistical analyses

A repeated-measures ANOVA was conducted to examine changes in symptoms from pre- to posttreatment. The proportion of missing data was 10.5%. To address missing data, the expectation maximization (EM) method in SPSS, version 29, was utilized to replace missing values. The use of the EM algorithm is appropriate when less than 25% of data are missing and the missing data are deemed to be missing at random, which was confirmed to be the case for the present dataset (Little`s MCAR test *X²* (18.798), *df* = 17, *p* = .340).

## Results

### Demographic and clinical characteristics

**Fig. 1:**
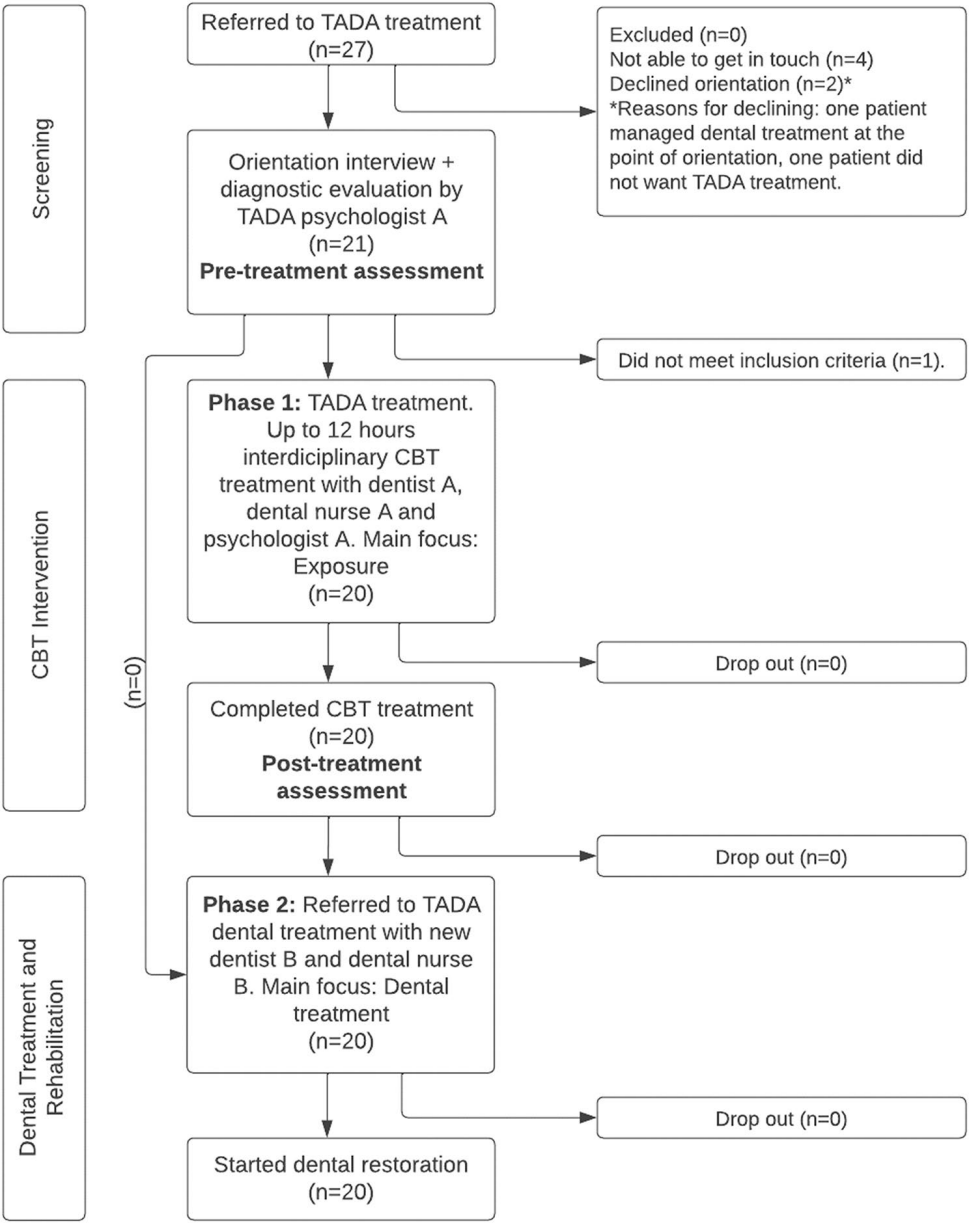
The flow of TADA treatment after referral

Twenty-seven patients were referred for TADA treatment during the designated trial period. Of these patients, we were unable to reach four patients on the waiting list to offer them an initial appointment. Furthermore, two patients declined treatment. Of these two patients, one had already managed dental treatment at the time of orientation, and the other did not want TADA treatment. One patient did not meet the inclusion criteria after treatment orientation and evaluation. Consequently, 20 consecutive patients referred to the regional TADA outpatient clinic for adults in the county of Møre and Romsdal, Norway, were included (please see Fig. 1 for the flow chart). Of these 20 patients, 12 were referred by oral health personnel (dentists, dental hygienists, oral surgeons), four were referred by general practitioners, two were referred by psychiatric services, and two referred themselves.

The mean time since the last dental treatment was 10.7 years (range = 0–30 years). The study participants had an average age of 41.8 years (range = 21–64 years), 75% were female, and 65% were married or cohabiting. A minority of patients had completed higher education, and half received social security benefits. A significant proportion of individuals (70%) stated that their personal finances, in part or significantly, had affected their ability to pursue dental treatment. Furthermore, the patients had been on a waiting list for a duration of 42 months prior to the start of phase 1 of the TADA treatment.

All patients in this sample met the diagnostic criteria for odontophobia, and all underwent interdisciplinary CBT treatment. No patients were referred directly to the TADA dentist team after treatment orientation. Furthermore, no patients were referred for trauma therapy before or during CBT treatment by the TADA teams. Two patients were granted additional exposure sessions (one and seven sessions).

Ten patients reported that domestic violence and/or abuse experiences were the cause of their dental anxiety. Of the other ten patients, three patients did not report traumatic incidents, while seven did not relate their abuse/violence experiences as the cause, or sustaining cause, of their odontophobia. None of the patients stated that they were survivors of torture experiences. 70% reported a history of sexual abuse, as measured by the stressful life event questionnaire. Furthermore, 65% reported exposure to violence in close relationships. 55% reported being survivors of both sexual abuse and violence in close relationships. Patients exposed to potential stressful life events reported a mean of 6.3 (range = 3–11) potential traumatic experiences.

70% of patients reported having comorbid psychiatric disorders, and six (30%) patients simultaneously received general mental health treatment. Patients did not have to end their ongoing treatments to be included in the study. The most prevalent comorbid diagnoses were mood disorders (35%), attention-deficit/hyperactive disorder (30%), and posttraumatic stress disorder (30%). Table 1 summarizes the sample’s characteristics.

**Table 1:** Demographics and clinical characteristics of the TADA patient group (*n* = 20)

Variable	M(SD)	n(%)
Age	41.8 (11.0)	
Female sex		15 (75%)
Married/Cohabiting		13 (65%)
Higher education		3 (15%)
Personal economic status affected dental treatment execution		14 (70%)
Years since last dental treatment	10.7 (9.8)	
Met the diagnostic criteria for odontophobia		20 (100%)
Odontophobia associated with abuse or violence		10 (50%)
Odontophobia unrelated to abuse or violence		10 (50%)
Previous negative experiences with dental treatment		13 (65%)
Number of stressful life events (SLESQ)	6.3 (2.6)	
Comorbid psychiatric diagnoses		14 (70%)
Mood Disorders		7 (35%)
ADHD		6 (30%)
PTSD		6 (30%)
Other comorbid disorders		9 (45%)
Referred for general anaesthesia during CBT		2 (10%)
Total number of CBT treatment sessions	10.8 (2.6)	
Dental attendance posttreatment		20 (100%)

*Note* CBT = Cognitive–behavioural therapy; SLESQ = Stressful Life Events Screening Questionnaire; ADHD = Attention-deficit/hyperactivity disorder; PTSD = Posttraumatic stress disorder; Other comorbid disorders = Eating disorder, anxiety disorder, autism spectrum diagnosis, borderline personality disorder

There were no dropouts during the interdisciplinary CBT phase of the TADA treatment program. On average, patients received 10.8 interdisciplinary CBT sessions (SD = 2.6, range = 6–19 sessions). All patients were referred to the TADA dentist team following the completion of the CBT intervention. Additionally, all patients attended further dental appointments and initiated dental treatment and rehabilitation.

### Changes in symptoms

There was a significant reduction in the symptoms of dental anxiety from pre- to post-treatment as measured with the MDAS (λ = 0.07, *F*(1,19) = 262.10, *p* < .001, *d* = 3.07). There was also a significant reduction in symptoms of dental fear as measured with the DFS (λ = 0.25, *F*(1,19) = 57.36, *p* < .001, *d =* 2.18).

For the 17 patients who reported having traumatic experiences, there were large reductions in symptoms of post-traumatic stress as measured with the PCL-5 (λ = 0.56, *F*(1,16) = 12.43, *p* = .003, *d* = 3.04). For the whole sample, there was an improvement in symptoms of depression as measured with the PHQ-9 (λ = 0.50, *F*(1,19) = 19.36, *p* < .001, *d* = 1.00), and there were moderate improvements in symptoms of generalized anxiety as measured with the GAD-7 (λ = 0.74, *F*(1,19) = 6.60, *p* < .001, *d* = 0.57). A summary of the analyses is displayed in Table 2.

**Table 2:** Results (*M* and *SD*) for the primary and secondary outcome measures (*N* = 20)

Variable	Pre	Post	Lambda	F	*p*	d
MDAS	22.0 (3.5)	11.3 (3.5)	0.068	262.10	<0.001	3.07
DFS	83.1 (14.4)	50.1 (15.7)	0.249	57.36	<0.001	2.18
GAD-7	10.4 (5.2)	7.4 (5.2)	0.742	6.60	< 0.019	0.57
PHQ-9	12.3 (5.2)	7.3 (4.9)	0.495	19.36	<0.001	1.00
PCL-5*	41.7 (2.9)	29.3 (5.0)	0.563	12.425	< 0.003	3.04

*Note* MDAS = The Modified Dental Anxiety Scale; DFS = Dental Fear Survey; GAD-7 = Generalized Anxiety Disorder-7; PHQ-9 = Patient Health Questionnaire-9; PCL-5 = PTSD Checklist for DSM-5. d = Cohen’s*d (M*_*pre*_*– M*_*post*_*)/SD*_pooled_* PCL-5 were only used for those who reported a history of traumatic experiences (*n* = 17)

Subgroup analyses were conducted to inspect possible effects of ongoing psychological treatment, and to compare possible differences between patients with and without a history of abuse. The results are summarized in supplemental Table S1. There were no associations between ongoing psychological treatment and changes in MDAS and DFS. However, patients with ongoing psychological treatment showed less improvement in symptoms of depression and anxiety. Patients with a history of abuse reported similar changes in symptoms as patients without such history.

### Oral health and dental treatment needs

The average DMFT score for the total sample was 18.8 (range 10–36). The patients in the sample had on average 6.6 decayed teeth, 5.6 missing teeth and 6.7 filled teeth. See Table 3 for the total average DMFT score and MPS. On average, patients had an MPS of 2.8 (range 2–6).

**Table 3:** DMFT and MPS for the total sample

		Mean	SD	Range
DMFT	Total	18.8	5.5	10–36
	Decayed	6.6	5.5	1–24
	Missing	5.6	3.7	0–13
	Filled	6.7	3.5	1–14
MPS		2.8	1.2	2–6

*Note* DMFT = Decayed, Missing and Filled Teeth; MPS = Mucosal and Plaque Score

## Discussion

The present study aimed to evaluate the implementation of integrated psychological and dental treatment within the TADA team for a sample of patients exposed to traumatic events and/or diagnosed with odontophobia. Overall, the sample reported positive treatment outcomes. Notably, no patients declined further dental treatment after the CBT intervention, indicating that the treatment was both accepted and tolerated by the participants.It is promising that all patients in this sample completed the interdisciplinary CBT treatment intervention despite their previous psychological trauma experiences, high degree of psychiatric comorbidities, prolonged dental avoidance behaviour, and the absence of anxiolytic drug administration. Additionally, all patients were referred to and started dental treatment and rehabilitation. These results suggest that the treatment approach was acceptable for vulnerable patients with a history of traumatic experiences and patients with odontophobia. This finding is significant given that the implementation of TADA teams is unique, the service has not been evaluated, and characteristics of the specific patient group have not been described in detail [28].

There were large and significant improvements in all measures of dental fear and phobia after CBT treatment. However, some studies indicated that a relatively large proportion of patients do not show improved dental attendance despite reporting reductions in their dental anxiety level following different treatments [47]. Our findings are align more closely with a previous meta-analysis on behavioural interventions for dental fear in adults, showing medium to large effect sizes for self-reported dental anxiety after behavioural interventions and post-treatment attendance at dental visits with rates between 33% and 100% within 6 months after treatment [25]. All patients initiated dental treatment, but the study lacks information concerning long-term dental care attendance. Additionally, consistent with other research indicating wider positive life changes after CBT for dental anxiety treatment, our study found decreased symptoms of depression and generalized anxiety following treatment [48, 49].

Most patients in our sample had a history of being exposed to potentially traumatic life experiences and had a high prevalence of comorbid psychiatric diagnoses. The significant reduction in posttraumatic stress symptoms suggest that the treatment was well tolerated and could alleviate PTSD symptoms. Although the treatment did not have a direct focus on altering the primary traumatic experience and related psychopathology, the treatment intervention did focus on managing trauma symptoms as presented in the dental care setting. The purpose of this was to make it possible for the patients to have new and corrective learning experiences with dental treatment and to alter dental-related catastrophic thoughts and behaviours. These results are thus in line with research that indicates that the exposure of patients to corrective information that violates their expectations is central to fear reduction in psychological therapy [50]. Furthermore, these results support previous findings from qualitative studies of trauma-informed treatment interventions and indicate that interdisciplinary CBT could be potentially beneficial and feasible for patients exposed to psychological trauma caused and/or maintained by reasons other than previous dental treatment experiences [20, 21, 51].

The patients included in this study had a formal diagnosis of dental phobia at treatment entry and had avoided dental treatment for over a decade. The longevity of dental avoidance in our sample was concordant with other findings [25, 52]. In summary, we found significant variations in oral health and dental treatment needs as measured by the total MPS and DMFT score. Dental treatment needs were heterogeneous, varying between no/little to many dental treatment needs. We found that the dental status of the sample was in line with a previous study on treatment-seeking patients with odontophobia in Norway [13] and Sweden [14]. The Norwegian study found a DMFT mean score of 16.4 in their sample while the Swedish study found an average DMFT score of 18.6, compared to 18.8 in the current study. We also found significant variations in oral health as measured by the total MPS. This is also in line with the previous studies on dental status in treatment-seeking odontophobia patients in Norway [13] and Sweden [14]. The variations in the MPS reflect that some patients had a reduced ability for dental-related self-care behaviour, while others had an intact ability to take care of their own oral health despite severe dental anxiety.

Most patients reported having a low socioeconomic background, which could be associated with a heightened risk of dental fear [53]. Many patients in the sample (70%) stated that their personal economic status, in part or significantly, had affected their ability to receive dental treatment. These findings suggest that a considerable number of patients in the TADA intervention would have faced financial constraints, making it unlikely for them to independently pursue dental treatment due to limited financial resources. The fact that the TADA treatment (both CBT and dental treatment and rehabilitation) was delivered free of charge, therefore, appears to have been important for patients to be able to overcome their dental treatment difficulties. The availability of affordable treatment could play an important role in facilitating access to necessary dental treatment interventions for these patients.

Interdisciplinary CBT treatment was given. Due to limited resources, oral health care personnel are often required to provide anxiety treatment without access, or with limited access, to psychological expertise. The findings in this study suggest that mental health professionals could be a valuable allies for oral health care personnel.

The current case series study must be considered in light of several limitations. The small number of participants and the lack of a control condition makes it impossible to determine whether the findings are unique to TADA treatment and to evaluate the relative efficacy of the treatment received. The study also lacked a long-term follow-up assessment. Furthermore, some patients with dental fear have been subjected to torture [54]; however, such experiences were not reported by the current sample, making it difficult to generalize the findings to patients with a history of torture. The study also lacked information about substance abuse and previous negative experiences with dental care.

## Conclusions

This study indicates that interdisciplinary CBT in the context of TADA teams could be both beneficial and acceptable for odontophobia and dental anxiety related to sexual abuse and violence. The results suggest that mental health professionals could be important allies for oral health professionals when caring for patients with severe dental anxiety and odontophobia. System-oriented interventions could benefit from interdisciplinary collaboration, striving to offer seamless and effective treatment options to vulnerable patient populations. A larger controlled study examining the long-term effects of TADA treatment is warranted.

## Electronic supplementary material

Below is the link to the electronic supplementary material.

Supplementary Material 1

## Data Availability

The anonymized datasets used during the current study are available from the corresponding author upon reasonable request.

## Abbreviations

CBT: Cognitive-Behavioural Therapy
TADA: Torture, Abuse, and Dental Anxiety
PTSD: Posttraumatic stress disorder
MPS: Mucosal and Plaque Score
DMFT: Decayed, Missing, and Filled Teeth
MDAS: The Modified Dental Anxiety Scale
DFS: Dental Fear Survey
GAD-7: Generalized Anxiety Disorder-7
PHQ-9: Patient Health Questionnaire-9
PCL-5: PTSD Checklist for DSM-5

## References

[1] Kisely S, Sawyer E, Siskind D, Lalloo R. The oral health of people with anxiety and depressive disorders: a systematic review and meta-analysis. J Affect Disord. 2016;200:119–32. 27130961 10.1016/j.jad.2016.04.040

[2] Choi J, Price J, Ryder S, Siskind D, Solmi M, Kisely S. Prevalence of dental disorders among people with mental illness: an umbrella review. Aust N Z J Psychiatry. 2022;56(8):949–63. 34461748 10.1177/00048674211042239

[3] Torales J, Barrios I, González I. Oral and dental health issues in people with mental disorders. Medwave. 2017;17(8):e7045. 28937973 10.5867/medwave.2017.08.7045

[4] Turner E, Berry K, Aggarwal VR, Quinlivan L, Villanueva T, Palmier-Claus J. Oral health self-care behaviours in serious mental illness: a systematic review and meta-analysis. Acta Psychiatr Scand. 2022;145(1):29–41. 33862664 10.1111/acps.13308

[5] Yazdanian M, Armoon B, Noroozi A, Mohammadi R, Bayat AH, Ahounbar E, Higgs P, Nasab HS, Bayani A, Hemmat M. Dental caries and periodontal disease among people who use drugs: a systematic review and meta-analysis. BMC Oral Health. 2020;20(1):44. 32041585 10.1186/s12903-020-1010-3 PMC7011515

[6] Bjørkvik J, Quintero DPH, Vika ME, Nielsen GH, Virtanen JI. Barriers and facilitators for dental care among patients with severe or long-term mental illness. Scand J Caring Sci. 2022;36(1):27–35. 33523487 10.1111/scs.12960 PMC9292278

[7] Kani E, Asimakopoulou K, Daly B, Hare J, Lewis J, Scambler S, Scott S, Newton JT. Characteristics of patients attending for cognitive behavioural therapy at one UK specialist unit for dental phobia and outcomes of treatment. Br Dent J. 2015;219(10):501–6. discussion 506. 26611310 10.1038/sj.bdj.2015.890

[8] Locker D, Liddell A, Dempster L, Shapiro D. Age of onset of dental anxiety. J Dent Res. 1999;78(3):790–6. 10096455 10.1177/00220345990780031201

[9] Nermo H, Willumsen T, Rognmo K, Thimm JC, Wang CEA, Johnsen JK. Dental anxiety and potentially traumatic events: a cross-sectional study based on the Tromsø Study-Tromsø 7. BMC Oral Health. 2021;21(1):600. 34814891 10.1186/s12903-021-01968-4 PMC8609887

[10] Hakeberg M, Berggren U, Gröndahl HG. A radiographic study of dental health in adult patients with dental anxiety. Community Dent Oral Epidemiol. 1993;21(1):27–30. 8432101 10.1111/j.1600-0528.1993.tb00714.x

[11] Schuller AA, Willumsen T, Holst D. Are there differences in oral health and oral health behavior between individuals with high and low dental fear? Community Dent Oral Epidemiol. 2003;31(2):116–21. 12641592 10.1034/j.1600-0528.2003.00026.x

[12] Halonen H, Nissinen J, Lehtiniemi H, Salo T, Riipinen P, Miettunen J. The association between dental anxiety and psychiatric disorders and symptoms: a systematic review. Clin Pract Epidemiol Ment Health. 2018;14:207–22. 30288171 10.2174/1745017901814010207 PMC6142663

[13] Agdal ML, Raadal M, Skaret E, Kvale G. Oral health and oral treatment needs in patients fulfilling the DSM-IV criteria for dental phobia: possible influence on the outcome of cognitive behavioral therapy. Acta Odontol Scand. 2008;66(1):1–6. 18320411 10.1080/00016350701793714

[14] Bohman W, Lundgren J, Berggren U, Carlsson S. Psychosocial and dental factors in the maintenance of severe dental fear. Swed Dent J. 2010;34(3):121. 21121411

[15] Lenk M, Berth H, Joraschky P, Petrowski K, Weidner K, Hannig C. Fear of dental treatment–an underrecognized symptom in people with impaired mental health. Dtsch Arztebl Int. 2013;110(31–32):517–22. 24069071 10.3238/arztebl.2013.0517 PMC3782017

[16] Leeners B, Stiller R, Block E, Görres G, Imthurn B, Rath W. Consequences of childhood sexual abuse experiences on dental care. J Psychosom Res. 2007;62(5):581–8. 17467413 10.1016/j.jpsychores.2006.11.009

[17] Dougall A, Fiske J. Surviving child sexual abuse: the relevance to dental practice. Dent Update. 2009;36(5):294–6. 303 – 294. 19585853 10.12968/denu.2009.36.5.294

[18] Levine PA. Waking the tiger: Healing trauma: the innate capacity to transform overwhelming experiences. North Atlantic Books; 1997.

[19] Larijani HH, Guggisberg M. Improving Clinical Practice: What Dentists Need to Know about the Association between Dental Fear and a History of Sexual Violence Victimisation. *Int J Dent* 2015, 2015:452814. 10.1155/2015/452814 PMC4309219 25663839

[20] Fredriksen TV, Søftestad S, Kranstad V, Willumsen T. Preparing for attack and recovering from battle: understanding child sexual abuse survivors’ experiences of dental treatment. Community Dent Oral Epidemiol. 2020;48(4):317–27. 32436226 10.1111/cdoe.12536

[21] Kranstad V, Søftestad S, Fredriksen TV, Willumsen T. Being considerate every step of the way: a qualitative study analysing trauma-sensitive dental treatment for childhood sexual abuse survivors. Eur J Oral Sci. 2019;127(6):539–46. 31731327 10.1111/eos.12661

[22] Stalker CA, Russell BDC, Teram E, Schachter CL. Providing dental care to survivors of childhood sexual abuse: treatment considerations for the practitioner. J Am Dent Association. 2005;136(9):1277–81. 10.14219/jada.archive.2005.0344 16196233

[23] Norwegian Directorate of Health. Tilrettelagte tannhelsetilbud for mennesker som er blitt utsatt for tortur, overgrep eller har odontofobi (facilitated dental health services for people who have been subjected to torture, abuse or odontophobia). Oslo: Helsedirektoratet (Norwegian Directorate of Health); 2010.

[24] Gordon D, Heimberg RG, Tellez M, Ismail AI. A critical review of approaches to the treatment of dental anxiety in adults. J Anxiety Disord. 2013;27(4):365–78. 23746494 10.1016/j.janxdis.2013.04.002

[25] Kvale G, Berggren U, Milgrom P. Dental fear in adults: a meta-analysis of behavioral interventions. Community Dent Oral Epidemiol. 2004;32(4):250–64. 15239776 10.1111/j.1600-0528.2004.00146.x

[26] Boman UW, Carlsson V, Westin M, Hakeberg M. Psychological treatment of dental anxiety among adults: a systematic review. Eur J Oral Sci. 2013;121(3 Pt 2):225–34. 23659254 10.1111/eos.12032

[27] De Jongh A, Van Der Burg J, Van Overmeir M, Aartman I, Van Zuuren FJ. Trauma-related sequelae in individuals with a high level of dental anxiety. Does this interfere with treatment outcome? Behav Res Ther. 2002;40(9):1017–29. 12296487 10.1016/S0005-7967(01)00081-X

[28] Bryne E, Hean SCPD, Evensen KB, Bull VH. Exploring the contexts, mechanisms and outcomes of a torture, abuse and dental anxiety service in Norway: a realist evaluation. BMC Health Serv Res. 2022;22(1):533. 35459239 10.1186/s12913-022-07913-7 PMC9026053

[29] Sheehan D, Janavs J, Baker R, Harnett-Sheehan K, Knapp E, Sheehan M. Mini international neuropsychiatric interview. Tampa: University of South Florida; 1994.

[30] Goodman LA, Corcoran C, Turner K, Yuan N, Green BL. Stressful life events screening questionnaire. Washington, DC: US Department of Veterans Affairs; 2013.

[31] Weathers FW, Litz BT, Keane TM, Palmieri PA, Marx BP, Schnurr PP. The ptsd checklist for dsm-5 (pcl-5). Boston, MA: National Center for PTSD; 2013.

[32] Humphris GM, Morrison T, Lindsay SJ. The modified dental anxiety scale: validation and United Kingdom norms. Community Dent Health. 1995;12(3):143–50. 7584581

[33] Humphris GM, Dyer TA, Robinson PG. The modified dental anxiety scale: UK general public population norms in 2008 with further psychometrics and effects of age. BMC Oral Health. 2009;9:20. 19709436 10.1186/1472-6831-9-20 PMC2743651

[34] King K, Humphris G. Evidence to confirm the cut-off for screening dental phobia using the modified dental anxiety scale. Soc Sci Dent. 2010;1(1):21–8.

[35] Kleinknecht RA, Klepac RK, Alexander LD. Origins and characteristics of fear of dentistry. J Am Dent Assoc. 1973;86(4):842–8. 4511174 10.14219/jada.archive.1973.0165

[36] Kleinknecht RA, Thorndike RM, McGlynn FD, Harkavy J. Factor analysis of the dental fear survey with cross-validation. J Am Dent Assoc. 1984;108(1):59–61. 6582116 10.14219/jada.archive.1984.0193

[37] Goodman LA, Corcoran C, Turner K, Yuan N, Green BL. Assessing traumatic event exposure: general issues and preliminary findings for the stressful life events screening questionnaire. J Trauma Stress. 1998;11(3):521–42. 9690191 10.1023/A:1024456713321

[38] Wortmann JH, Jordan AH, Weathers FW, Resick PA, Dondanville KA, Hall-Clark B, Foa EB, Young-McCaughan S, Yarvis JS, Hembree EA, et al. Psychometric analysis of the PTSD checklist-5 (PCL-5) among treatment-seeking military service members. Psychol Assess. 2016;28(11):1392–403. 26751087 10.1037/pas0000260

[39] Spitzer RL, Kroenke K, Williams JB. Validation and utility of a self-report version of PRIME-MD: the PHQ primary care study. Primary care evaluation of mental disorders. Patient health questionnaire. JAMA. 1999;282(18):1737–44. 10568646 10.1001/jama.282.18.1737

[40] Spitzer RL, Kroenke K, Williams JB, Lowe B. A brief measure for assessing generalized anxiety disorder: the GAD-7. Arch Intern Med. 2006;166(10):1092–7. 16717171 10.1001/archinte.166.10.1092

[41] Beard C, Björgvinsson T. Beyond generalized anxiety disorder: psychometric properties of the GAD-7 in a heterogeneous psychiatric sample. J Anxiety Disord. 2014;28(6):547–52. 24983795 10.1016/j.janxdis.2014.06.002

[42] Henriksen BM, Ambjørnsen E, Axéll TE. Evaluation of a mucosal-plaque index (MPS) designed to assess oral care in groups of elderly. Spec Care Dentist. 1999;19(4):154–7. 10765880 10.1111/j.1754-4505.1999.tb01378.x

[43] Broadbent JM, Thomson WM. For debate: problems with the DMF index pertinent to dental caries data analysis. Community Dent Oral Epidemiol. 2005;33(6):400–9. 16262607 10.1111/j.1600-0528.2005.00259.x PMC1388190

[44] Marthaler TM. Changes in dental caries 1953–2003. Caries Res. 2004;38(3):173–81. 15153686 10.1159/000077752

[45] Nadanovsky P, Sheiham A. Relative contribution of dental services to the changes in caries levels of 12-year-old children in 18 industrialized countries in the 1970s and early 1980s. Community Dent Oral Epidemiol. 1995;23(6):331–9. 8681514 10.1111/j.1600-0528.1995.tb00258.x

[46] Pandolfo I, Mazziotti S. OPT in Post-treatment Evaluation. In: *Orthopantomography.* edn. Milano: Springer Milan; 2013: 165–198.

[47] Aartman IH, de Jongh A, Makkes PC, Hoogstraten J. Dental anxiety reduction and dental attendance after treatment in a dental fear clinic: a follow-up study. Community Dent Oral Epidemiol. 2000;28(6):435–42. 11106016 10.1034/j.1600-0528.2000.028006435.x

[48] Vermaire JH, De Jongh A, Aartman IH. Dental anxiety and quality of life: the effect of dental treatment. Community Dent Oral Epidemiol. 2008;36(5):409–16. 18924257 10.1111/j.1600-0528.2007.00416.x

[49] Hakeberg M, Berggren U, Carlsson SG, Gröndahl HG. Long-term effects on dental care behavior and dental health after treatments for dental fear. Anesth Prog. 1993;40(3):72–7. 7645792 PMC2148740

[50] Craske MG, Treanor M, Conway CC, Zbozinek T, Vervliet B. Maximizing exposure therapy: an inhibitory learning approach. Behav Res Ther. 2014;58:10–23. 24864005 10.1016/j.brat.2014.04.006 PMC4114726

[51] Erga AH, Kvernenes KV, Evensen KB, Vika ME. Behandling av odontofobi for pasienter med post-traumatiske plager: en litteraturoversikt (treatment of dental phobia in patients with post-traumatic symptoms: a literature review). Nor Tann Tid. 2017;127(8):682–6.

[52] Willumsen T, Vassend O, Hoffart A. A comparison of cognitive therapy, applied relaxation, and nitrous oxide sedation in the treatment of dental fear. Acta Odontol Scand. 2001;59(5):290–6. 11680648 10.1080/000163501750541156

[53] Armfield JM, Spencer AJ, Stewart JF. Dental fear in Australia: who’s afraid of the dentist? Aust Dent J. 2006;51(1):78–85. 16669482 10.1111/j.1834-7819.2006.tb00405.x

[54] Høyvik AC, Willumsen T, Lie B, Hilden PK. The torture victim and the dentist: the social and material dynamics of trauma re-experiencing triggered by dental visits. J Rehabil Torture Vict Prev Torture. 2021;31(3):70–83.

