# Supplementary material for: Interdisciplinary CBT treatment for patients with odontophobia and dental anxiety related to psychological trauma experiences: a case series
Source: BMC Psychiatry. 2024 Sep 10;24:606. doi: 10.1186/s12888-024-06055-w (PMC11389481; doi:10.1186/s12888-024-06055-w)
Supplement: Supplementary file 1 — Supplementary Material 1 [file 12888_2024_6055_MOESM1_ESM.docx]

**Supplemental Table S1**

Changes in symptoms for subgroups (patients with concurrent psychological treatment and patients with dental anxiety related to abuse)

| Variable | Pre-treatment | | | | | | Post-treatment | | | | | | | | Time*group | | | | |  |  |
| --- | --- | --- | --- | --- | --- | --- | --- | --- | --- | --- | --- | --- | --- | --- | --- | --- | --- | --- | --- | --- | --- |
|  | *No psych. tx* | | | *Psych tx.* | | | *No psych tx.* | | | | *Psych tx.* | | | |  | | |  | |  |  |
|  | *M* | *SD* | | *M* | | *SD* | *M* | | | *SD* | *M* | | *SD* | | *F* | | | *p* | |  |  |
| MDAS | 21.1 | 3.8 | | 24.0 | | 1.3 | 10.6 | | | 3.2 | 12.8 | | 4.2 | | 0.20 | | | .657 | |  |  |
| DFS | 79.9 | 15.9 | | 90.5 | | 6.0 | 49.0 | | | 16.3 | 52.7 | | 15.4 | | 0.53 | | | .477 | |  |  |
| GAD-7 | 10.8 | 5.7 | | 9.4 | | 4.2 | 6.1 | | | 5.4 | 10.3 | | 3.7 | | 6.21 | | | .023 | |  |  |
| PHQ-9 | 12.7 | 5.8 | | 11.5 | | 3.8 | 5.9 | | | 4.9 | 10.7 | | 2.8 | | 8.30 | | | .010 | |  |  |
|  |  | | |  | | |  | | | |  | | | |  | | |  | |  |  |
|  | *No abuse* | | | *Abuse* | | | *No abuse* | | | | *Abuse* | | | |  | | |  | |  |  |
| MDAS | 21.5 | 3.8 | | 22.5 | | 3.2 | 11.9 | | | 3.4 | 10.7 | | 3.7 | | 3.06 | | | .097 | |  |  |
| DFS | 79.5 | 18.6 | | 86.6 | | 8.1 | 54.9 | | | 17.1 | 45.3 | | 13.4 | | 4.33 | | | .052 | |  |  |
| GAD-7 | 9.7 | 5.1 | | 11.1 | | 5.5 | 6.8 | | | 5.3 | 8.0 | | 5.3 | | 0.01 | | | .940 | |  |  |
| PHQ-9 | 11.8 | 6.2 | | 12.9 | | 4.1 | 6.9 | | | 5.9 | 7.7 | | 3.9 | | 0.02 | | | .899 | |  |  |
|  |  |  |  |  |  |  |  |  |  |  |  |  |  |  | |  |  | |  |  |  |

*Note*. Psych. tx = psychological treatment; MDAS=The Modified Dental Anxiety Scale; DFS= Dental Fear Survey; GAD-7= Generalized Anxiety Disorder-7; PHQ-9= Patient Health Questionnaire-9.
